# Aspects of vascularization in Multi-Organ-Chips

**DOI:** 10.1186/1753-6561-7-S6-O6

**Published:** 2013-12-04

**Authors:** Katharina Schimek, Reyk Horland, Sven Brincker, Benjamin Groth, Ulrike Menzel, Ilka Wagner, Eva-Maria Materne, Gerd Lindner, Alexandra Lorenz, Silke Hoffmann, Mathias Busek, Frank Sonntag, Udo Klotzbach, Roland Lauster, Uwe Marx

**Affiliations:** 1 TU Berlin, Institute of Biotechnology, Faculty of Process Science and Engineering, 13355 Berlin, Germany; 2 Fraunhofer IWS Dresden, 01277 Dresden, Germany; 3 TissUse GmbH, 15528 Spreenhagen, Germany

## Background

Enormous efforts have been made to develop circulation systems for physiological nutrient supply and waste removal of *in vitro *cultured tissues. These developments are aiming for *in vitro *generation of organ equivalents such as liver, lymph nodes and lung or even multi-organ systems for substance testing, research on organ regeneration or transplant manufacturing. Initially technical perfusion systems based on membranes, hollow fibers or networks of micro-channels were used for these purposes. However, none of the currently available systems ensures long-term homeostasis of the respective tissue over months. This is caused by a lack of *in vivo*-like vasculature which leads to continuous accumulation of protein sediments and cell debris in the systems. Here, we demonstrate a closed and self-contained circulation system emulating the natural blood perfusion environment of vertebrates at tissue level.

## Material and methods

**Figure 1:**
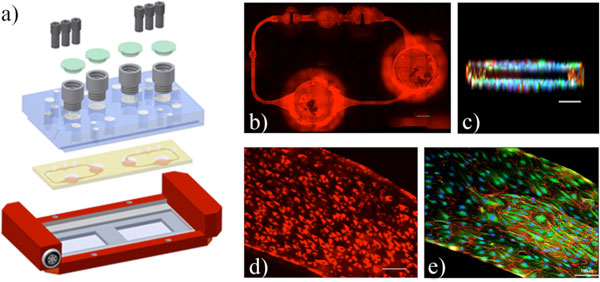
**HDMEC microvasculature in the MOC device**. **a) **Exploded view of the device comprising a polycarbonate CP (blue), the PDMS-glass chip accommodating two microvascular circuits (yellow; footprint: 76 mm × 25 mm; height: 3 mm) and a heatable MOC-holder (red). **b) **Calcein AM assay (red) showed viable and evenly distributed HDMEC in all areas of the circulation. Scale bar = 2 mm. **c) **Image stack taken by two-photon laser scanning microscopy. HDMEC were able to cover all walls of the channels forming a fluid tight layer. Functionality of the established microvascular vessel system was demonstrated by **d) **ac-LDL uptake of HDMEC and **e) **CD31 (red), vWF (green) expression throughout the entire cell population. Nuclei were counterstained with Hoechst 33342 (blue). Scale bar = 100μm.

The Multi-Organ-Chip (MOC) device accommodates two microvascular circuits (Figure 1a). Each circuit is operated by a separate peristaltic on-chip micropump, modified from Wu and co-workers [1]. Microfluidic 3D channels were formed in PDMS by replica molding from master molds and were afterwards closed by bonding to a cover-slip by air plasma treatment. To retain PDMS hydrophilicity, channels were filled with culture medium immediately after sealing. To emulate the natural blood perfusion environment, human dermal microvascular endothelial cells (HDMEC) were used. The cells were seeded into the PDMS channels and adhered to all channel walls after subsequent static cultivation on each channel side. Afterwards cells were cultured up to 14 days in PDMS channels under pulsatile flow conditions.

## Results

A miniaturized circulation system has been established over a period of 14 days by fully covering all channels and surfaces of the MOC with human microvascular endothelial cells. By injecting 2 × 10^7 ^cells ml^-1 ^into the channels, a homogeneous distribution of cells throughout all channels was achieved (Figure 1b). During the following static incubation, cells adhered well to the air plasma treated channel walls. A peristaltic micro-pump was used to create culture medium circulation. After adaption to shear stress, HDMEC showed an elongation and alignment parallel to the flow direction. Three-dimensional reconstitutions of image stacks indicate that cells formed confluent monolayers on all walls of the channels (Figure 1c). During the whole cultivation time they maintained adherence to the channel walls and were positive for Calcein AM viability staining (Figure 1b). After 14 days of culture HDMEC forming the microvascular circuit were positive for ac-LDL uptake (Figure 1d) and expressed the endothelial-specific marker CD31 and von Willebrand Factor (vWF) (Figure 1e).

## Conclusion

A robust procedure applying pulsatile shear stress has been established to cover all fluid contact surfaces of the system with a functional, tightly closed layer of HDMEC.

Long-term cultivation of elongated and flow-aligned HDMEC inside the chip-based microcirculation was demonstrated over a period of 14 days. For such endothelialized microfluidic devices to be useful for substance testing, it is essential to show long-term viability and function in the presence of physiological flow rates as shown here. These artificial vessels are an important approach for systemic substance testing in Multi-Organ-Chips. The miniaturized circulation system creates the conditions for circulation of nutrients through the organoid culture chamber, allows for *in vivo*-like crosstalk between endothelial cells and tissues and prevents clumping inside the channels. Compared with conventional cell culture techniques, a microfluidic-based cell culture may mimic more accurate *in vivo*-like extracellular conditions, as the culture of cells and organ models in perfused microfluidic systems can improve their oxygen and nutrient supply. This makes it suitable for long-term cultivation and more efficient drug studies. In future, such endothelialized bioreactors might be used for testing vasoactive substances. Finally, the described system can now be used for the establishment of organ-specific capillary networks. Here, we will adhere to our recently published roadmap toward vascularized ''human-on-a-chip'' models to generate systemic data fully replacing the animals or human beings currently used [2].
